# Quantitative Evaluation of Contrast-Enhanced Ultrasonography in the Diagnosis of Chronic Ischemic Renal Disease in a Dog Model

**DOI:** 10.1371/journal.pone.0070337

**Published:** 2013-08-01

**Authors:** Yi Dong, Wenping Wang, Jiaying Cao, Peili Fan, Xiyuan Lin

**Affiliations:** Department of Ultrasound, Zhongshan Hospital, Fudan University, Shanghai, China; University of Sao Paulo Medical School, Brazil

## Abstract

**Objectives:**

The aim of this feasibility study was to prospectively explore in a dog model of chronic ischemic renal disease (CIRD) the hypothesis that real-time contrast-enhanced ultrasonography (CEUS) can quantitatively evaluate the early perfusion changes of renal cortex.

**Materials and Methods:**

In this animal care and use committee-approved study, the model of CIRD was carried out in healthy dogs (10.0∼12.0 kg, n = 5), by placing the Ameroid ring constrictors on the distal portion of right renal artery through operation. CEUS monitoring of right kidney perfusion was performed by intravenous bolus injection of 0.6 ml Sulfur hexafluoride filled microbubbles (SonoVue; Bracco S.P.A., Milan, Italy) every week after operation. The slope rate of ascending curve (A) and descending curve (α), area under curve (AUC), derived peak intensity (DPI), and time to peak (TTP) were measured in renal cortex using commercial quantification software (Q-LAB version 6; Philips Medical Systems, Bothell,WA,USA). The sensitivity of CEUS was compared with blood serum urea nitrogen (BUN) and serum creatinine (SCr) level.

**Results:**

With the progression of CIRD, dogs showed delayed enhancement and perfusion in renal CEUS curve. Earliest significant changes happened 4 weeks after operation on DPI and TTP which changed from 13.04±2.71 to 15.58±4.75 dB and 9.03±2.01 to 10.62±6.04 sec, respectively (*P*<.05).

**Conclusions:**

CEUS can display the perfusion changes of CIRD in the early period.

## Introduction

The rapid technological advances over the past decade have been followed by the introduction of real-time contrast-enhanced ultrasonography (CEUS), which has significantly extended the usefulness of ultrasound imaging in human medicine and medical research. Ultrasound contrast agents (USCAs) allow the development of new functional applications for renal blood flow quantification [1]. New software tools make it possible to quantitatively evaluate the tissue enhancement and describe important parameters of tissue vitality.

Chronic ischemic renal disease (CIRD) which is very common in clinical medicine is very complicated in its pathological process. Impairment of perfusion is an early event in the course of renal dysfunction and usually precedes functional impairment [2]. The ability to accurately measure variations in renal perfusion could provide important clinical insights into renal function. Consequently, assessment of perfusion in the early period of CIRD is of the utmost importance [3]. Various noninvasive imaging methods have been used to evaluate renal dysfunction of CIRD. Unfortunately, they are not sensitive in the early stage, and data from different imaging methods are variable, thereby leading to difficulty in diagnosing early renal dysfunction [4], [5].

CIRD is characterized by a gradual decline of glomerular filtration rate (GFR). Currently, nuclear renogram, computed tomography, and magnetic resonance imaging are the most common imaging tests to quantitatively assess renal perfusion. However, they have significant limitations for quantifying renal blood flow in CIRD: they are either invasive, related to the use of ionizing radiation, highly costly, rely on the use of tracers that are diffusible or have kinetics that are complicated by tubular transport or glomerular filtration [2], [4].

As a safe and readily available method, gray scale US is widely used in clinical practice. However, early stage of CIRD is not reflected in specific changes in renal morphology. Color and spectral Doppler are the currently used methods to non-invasively assess renal perfusion. Their diagnostic values are still debatable because of their inherent limitations, such as the low sensitivity in slow flow and angle dependency [6]. Since USCAs have become available for the first time, functional studies with ultrasound have been made possible. Because microbubbles remain entirely within the intravascular space, have a rheology similar to that of red blood cells and are not affected by glomerular filtration or tubular transport, it may be possible to estimate blood flow by quantifying the contrast enhancement [7]–[8]. Unlike many other techniques, USCAs tolerance in clinical practice is excellent, no renal toxicity has been reported and microbubbles are rapidly cleared from the circulation after administration. The administration of USCAs can be repeated even in patients with renal failure in a very short time. Sehgal et al [9] first reported renal perfusion imaging with a sonographic contrast agent in 1998. Since then, many clinical investigation or experimental studies have shown that the use of microbubble agents in color and power Doppler sonography can improve the detection and characterization of various renal perfusion abnormalities [10]–[12]. However, major clinical applications of contrast ultrasound in the kidneys are related to the assessment of masses or regional perfusion differences, such as in pyelonephritis [13]. Changes in diffuse renal disease shown by CEUS are little explored or quantified.

More than 90% of total renal blood flow (RBF) entering the kidney, supplies the renal cortex; therefore a change in cortical microbubbles velocity was used as a surrogate for alteration in total RBF [8]. We hypothesized that measurements of their renal tissue kinetics could be used to quantify renal blood flow. As a significant, but non-specific reduction in renal perfusion is usually appreciable in CIRD, we hypothesized that changes in total CIRD would be mirrored by changes in cortical microbubble velocity during CEUS, which would provide more specific morphologic and functional information in diagnosing CIRD.

Thus, the purpose of our study was to prospectively test–in a dog model of chronic ischemic renal disease–the hypothesis that real-time contrast-enhanced ultrasonography can quantitatively evaluate the perfusion changes of renal cortex in the early stages of the disease.

## Materials and Methods

### Animal Model

This study was carried out in strict accordance with the recommendations in the guidelines issued by the National Institutes of Health for care of laboratory animals. The protocol was approved by the Committee on the Ethics of Animal Experiments of the Zhongshan Hospital, Fudan University (Permit Number: 08-1002). All surgery was performed under sodium pentobarbital anesthesia, and all efforts were made to minimize suffering. Use of the dogs in experimental models of kidney perfusion is well established. Five adult male healthy dogs (10.0∼12.0 kg of weight) were studied.

Food was withheld from each dog 12 hours before anesthesia. After general anesthesia induced by intravenous injection of 3% pentobarbital (1 ml kg^−1^), the dogs were fixed in a supine position. The hind leg veins of dogs were exposed and cannulated with 10F catheters for administration of microbubbles and fluids. The model of CIRD was carried out in dogs by operation. In all animals, the abdominal aorta, the right renal arteries and right kidneys were surgically exposed after midline laparotomy. Then, the Ameroid ring constrictors (AC, Research Instrument NW Co. Ltd. USA) were placed on the distal portion of the right main renal artery. The size of the AC was chosen to fit snugly around the right main renal artery without producing any initial constriction. The size of AC placed in 5 dogs was: a 5 mm AC in one dog, 4 mm AC in three dogs and a 3 mm AC in one dog.

After surgery, we continuously monitored dogs’ heart, vital signs and physiological parameters. We established humane endpoints by intravenous injection of diazepam.

### Protocol

In each dog, gray scale ultrasonography (US), color Doppler flow imaging (CDFI) and CEUS were performed for both kidneys before and every week after the operation. We observe the size of both kidneys, the location of ACs with gray scale US. And evaluate the CDFI of right renal interlobular arteries. Main renal artery occlusion was determined by US and CDFI at weekly intervals after AC placement.

CEUS renal perfusion images were converted into TIC. TIC was generated from the region of interest drawn over the anterior portion on the peripheral renal cortex, Quantitative perfusion parameters were acquired at each stage. At each time before CEUS procedure, blood samples were taken from hind leg veins by one individual. The sensitivity of CEUS in diagnosing CIRD was compared with blood serum urea nitrogen (BUN) µmol*l^−1^and serum creatinine concentration (SCr) level mmol*l^−1^.

### CEUS Examination

All CEUS procedures were performed by a sonographer, with 10 years of experience. Renal perfusion imaging with microbubble contrast agent was carried out on a Philips iU22 (Bothell, WA, USA) equipped with Qlab (version 6; Philips Medical Systems, Bothell, WA, USA) to analysis and display the time-intensity curves (TIC). A C5-2 convex transducer with a pulse repetition frequency (PRF) of 16 Hz was used. The acoustic power was set at the default setting. The mechanical index (MI) was set at 0.07, the gain was set at 90%, and the dynamic range was 66% to 72%. The USCAs (SonoVue, Bracco S.P.A., Milan, Italy) was administered by intravenous bolus (0.6 ml) injection via the hind leg vein catheter, followed by a 5 ml saline flush.

Before injection of the USCAs, we performed a gray scale ultrasound examination of the kidney and determined a maximum longitudinal scanning plane that included the entire kidney. Throughout the examination, we manually held the probe in the same scanning plane; all images were taken after the probe was positioned in the maximum longitudinal plane of the kidney. We set image capture time of 160 seconds after the intravenous bolus injection of contrast.

### Conventional US and CDFI Parameters

Conventional CDFI demonstrated blood distribution appearance in dogs’ right kidneys. After color positioning of the right main renal artery, the Doppler sample volume was positioned with the Doppler angle kept below 60° with respect to the long axis of the artery. The pulse repetition frequency was set to avoid aliasing, and the wall filter optimized as low as possible to detect slow diastolic flow.

Assessment of resistance and Doppler signals by CDFI were obtained from the same intralobular renal arteries in up pole of renal cortex. The peak systolic velocity (PSV) and resistance index (RI) were assessed by using six different measurements performed by a single investigator. Color gain was fixed at 80∼90, wall filter were at low level.

### Pathological Analysis

After the sacrifices of each dog, both kidneys and vessel inner AC were surgically removed and placed in a formalin solution for 12∼24 h. The kidneys were sectioned in the coronal plane (3 mm slice thickness).

Histological analysis after haematoxylin and eosin staining (original magnification from ×20 up to×60) was performed to confirm renal ischemic areas.

### Data Analyses

All real-time CEUS images were stored digitally on the hard disk as DICOM (Digital Image Communications in Medicine) images. All functional data were then transferred to a personal computer and blindly analyzed by a radiologist with 7 years of experience in abdominal CEUS. For each kidney, the renal perfusion images were analyzed for three times. Renal perfusion images were converted into TIC by Qlab quantification software, which was generated from the region of interest (ROI). ROI was drawn over the anterior portion on the peripheral renal cortex, in the same depth, with the same size and shape (5 mm*5 mm square).

**Figure 1:**
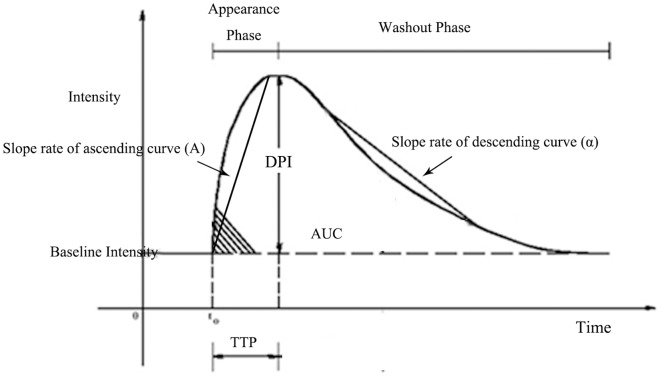
The gamma-variate function represents a suitable curve fit approximation of the time-intensity curve (TIC). With the formula for the gamma-variate function, quantitative perfusion parameters such as the slope rate of ascending curve (A), the slope rate of descending curve (α), area under curve (AUC), derived peak intensity (DPI), time to peak (TTP) were measured to estimate renal blood flow.

With a bolus injection of contrast agent, the shape of the TIC observed in the distal microcirculation resembles a highly skewed Gaussian curve with an exponentially decaying tail. With such indicator-dilution type of injections, the gamma-variate function represents a suitable curve fit approximation [14]. The formula for the gamma-variate function is as.
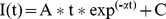
Where I(t) is the pixel intensity as a function of time, A is a scaling factor proportional to the rate of the initial upslope of the bolus wash-in, and α is a rate constant, which is the reciprocal of the curve width. From this equation the area under the curve (A/α^2^) and curve amplitude (A/α* exp^1^ ) can be derived. The time to the peak occurs at 1/α. At long values of time, the curve decays to an asymptotic value of C, which represents the baseline intensity. Quantitative perfusion parameters such as the slope rate of ascending curve (A), the slope rate of descending curve (α), area under curve (AUC), derived peak intensity (DPI), time to peak (TTP) were measured using TIC to estimate renal blood flow(Fig. 1).

### Statistical Analyses

Data are expressed as mean ± standard deviation. As each dog came with a sudden death or sacrificed at different time, the observation duration of each dog is different. Comparisons between quantitative indexes of different stages were performed using Random-effects GLS regression,which is specified for statistical analysis of irregular data. Comparisons between SCr and BUN at different stages were performed using Bonferroni test. All statistical analyses were performed with SPSS 11.0 software package (SPSS, version 11.0 Inc. Chicago, IL, USA). A difference was considered statistically significant with *P*<.05.

## Results

### Establishment of Animal Model

All dogs recovered from anesthesia and surgery without complications. No signs of pain or edema occurred in the abdomen related to vascular occlusion of main renal artery.

Two dogs came with sudden death 6 weeks after operation, one dog died in 12 weeks after operation and another dog in 13 weeks after operation. We sacrificed the last dog in 16 weeks after operation.

Histopathologic analysis showed complete occlusion was observed about 6 weeks after AC placement.

### Real-time Perfusion of Kidney

**Figure 2:**
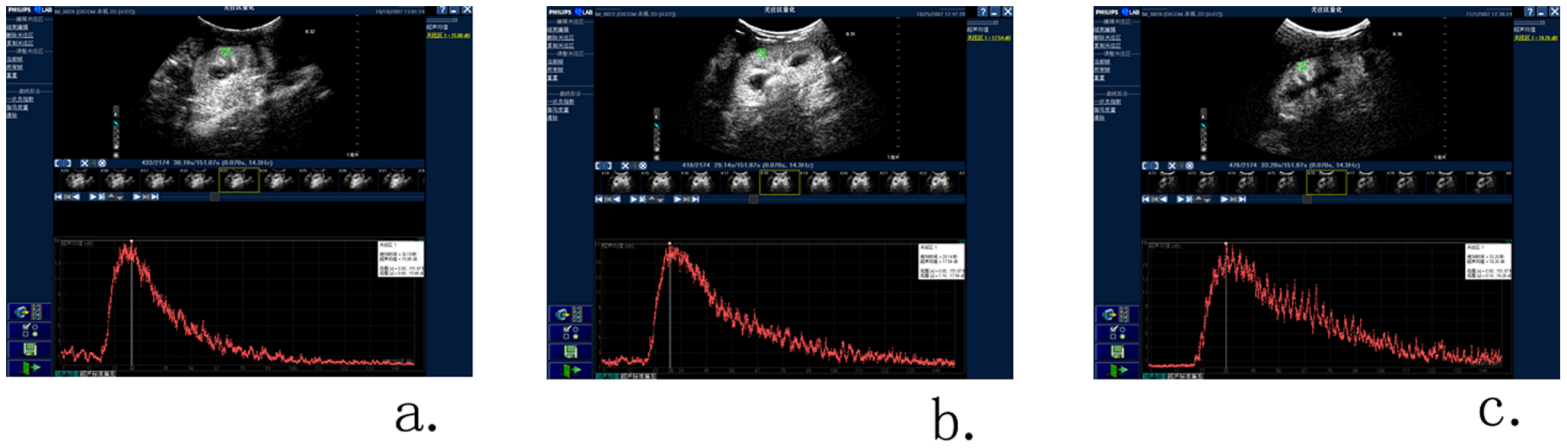
CEUS renal perfusion images. CEUS renal perfusion images were converted into a time-intensity curve (TIC) by Q-LAB quantification software, which was generated from the region of interest (ROI). (a) TIC of dog’s right kidney before operation. (b) TIC of dog’s right kidney 5 weeks after the placement of AC. (c) TIC of dog’s right kidney 7 weeks after the placement of AC. TIC showed weekly delayed enhancement and perfusion in the renal perfusion curve. The slope rate of ascending curve was lower and descending curve was higher. It took more time to reach the peak intensity.

After USCAs administration, in vivo gray scale showed real-time flow and perfusion in the kidney. We obtained renal perfusion imaging of both kidneys in all dogs. From the longitudinal plane, we observed a gradual enhancement from segmental renal arteries, small interlobular arteries in renal cortex to renal medullar arteries. CEUS renal perfusion images were then converted into TIC (Fig. 2). The slope rate of ascending curve was steep, and time to peak intensity was very short. Then the curve gradually descended to baseline. After the placement of AC, kidneys of these dogs showed weekly delayed enhancement and perfusion in the renal perfusion curve. The slope rate of ascending curve was lower and descending curve was higher. It took more time to reach the peak intensity (Fig. 2).

### Quantitative Renal Perfusion Data

**Figure 3:**
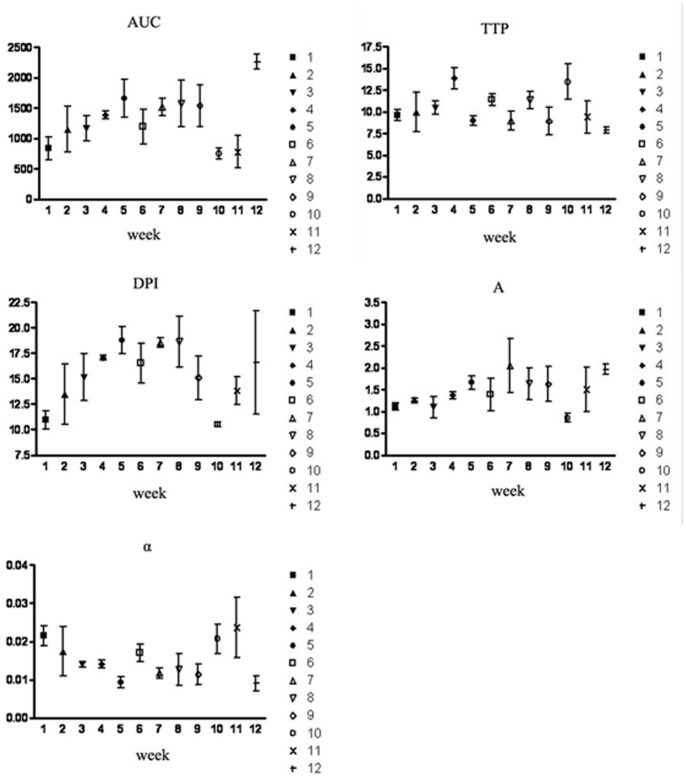
Changes of quantitative indexes in dogs’ CIRD models (1∼12 weeks after operation). With the progress of CIRD, area under curve (AUC), time to peak (TTP), derived peak intensity (DPI) and the slope rate of descending curve (α) gradually increased, however the slope rate of ascending curve (A)decreased.

With the progress of CIRD, quantitative indexes changed with times. AUC, TTP, DPI and α gradually increased, however A decreased (Fig. 3). Earliest significant changes happened 4 weeks after operation on DPI and TTP from 13.04±2.71 to 15.58±4.75 dB and 9.03±2.01 to 10.62±6.04 sec, respectively (*P*<.05). Significant changes happened 5 weeks on AUC, 7 weeks on A, and 11 weeks later on BUN and SCr (*P*<.05) (Table 1).

**Table 1:** Changes of quantitative indexes in dogs’ CIRD model.

CEUS Quantitative indexes	Weeks after operation	*P* value	95% Confidence range
**AUC**	5	0.020	[131.2631 1518.2]
**TTP**	4	0.032	[.3632358 8.106765]
**DPI**	4	0.043	[.1893859 11.96561]
**A**	7	0.028	[.1013517 1.773815]
**α**	5	0.014	[-.0218952 -.0024381]

CEUS = contrast-enhanced ultrasonography; AUC = area under curve; TTP = time to peak; DPI = derived peak intensity; A = slope rate of ascending curve; α = slope rate of descending curve.

### Conventional CDFI Parameters

After AC placement,weekly intervals with US showed ACs fixed tightly around distal portion of dogs’ right main renal arteries. CDFI indicate flow turbulence in main renal artery linked to ACs. A mosaic pattern was displayed by the ultrasound machine. Duplex spectral waveform showed irregular spectrum with increased PSV and RI in right main renal artery.

Conventional CDFI demonstrated no obviously perfusion change in both renal cortex. With the progression of CIRD, RI of right renal intralobular arteries gradually increased, while the PSV decreased. Significant changes were observed at 12 weeks (PSV) and 14 weeks (RI) after operation. (Table 2).

**Table 2:** Changes of PSV and RI of right renal interlobular arteries in dogs’ CIRD model.

CDFI indexes	Before operation	After operation	Weeks after operation	*P* value
**PSV(cm/s)**	70.91±6.68	49.00±7.36	12	**0.050**
**RI**	0.44±0.06	0.56±0.02	14	**0.048**

CDFI = color Doppler flow imaging; PSV = peak systolic velocity; RI = resistance index.

### Blood Tests and Histopathologic Analysis

Significant change happened 11 weeks later on BUN from 4.90±0.08 to 9.56±1.53 µmol*l^−1^ and SCr from 49.2±5.0 to 95.5±40.1 mmol*l^−1^ (*P*<.05).

Histopathologic analysis showed inflammation infiltration and fibrosis in right renal cortex, partial glomerular necrosis, denudation of renal tubular epithelial cell, apoptosis and focal necrosis. No significant change was observed in left kidney.

**Figure 4:**
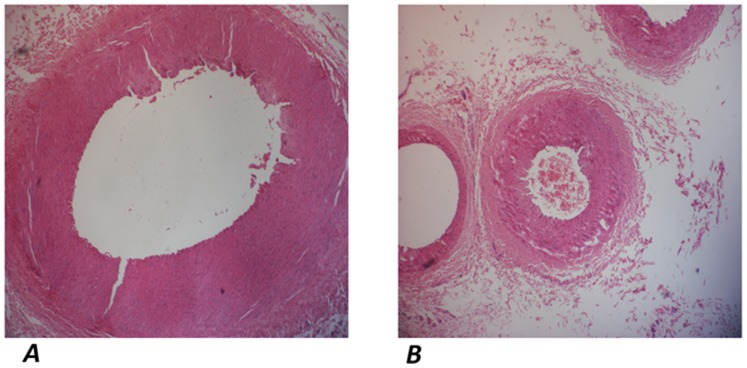
Pathologic changes of vessel inside and outside AC. (a) Vessel outside AC: slight hyperplasia of the vascular smooth muscle. (b) Vessel inside AC: Obvious hyperplasia of the vascular smooth muscle, narrowing of the renal artery with wall thickening. (hematoxylin and eosin, original magnification ×40).

Vessels inside AC narrowed with wall thickening. Different degrees of hyperplasia of the vascular smooth muscle inner AC were visible (hematoxylin and eosin, original magnification ×40) (Fig. 4).

## Discussion

Gradual occlusion techniques include the use of silk ligatures [15], perivascular cellophane bands [16], thrombogenic intravascular coils [17], and Ameroid ring Constrictor, which could produce progressive and permanent vascular attenuation [18]. In our research, we set up an animal model of CIRD by using the Ameroid ring constrictor. ACs were positioned within a ring of stainless steel, and then placed around the right renal arteries of dogs. After the AC is surgically placed around a blood vessel, the ameroid slowly absorbs fluid, expands and the diameter of the central cylindrical ring lumen gradually reduces in size [19]. It has been hypothesized that the complete vascular occlusion observed after placement of the AC on a vascular district may be the result of both physical expansion and an inflammatory response to the ameroid [19]–[20]. In our experiment, gradual occlusion of right renal arteries of dogs can be observed at weekly intervals. Main pathophysiologic changes after placement of AC were gradual vascular wall thickeness and decrease of luminal area. As a result, a gradual impairment of renal blood flow did occur 4∼6 weeks after operation.

The most interesting and promising recent development related to US appears to be the use of USCAs to plot contrast-distribution curves, known as TIC [10], [21]. Following intravenous bolus administration of USCAs, graphs are plotted that express signal intensity values, or contrast agent concentration, in the region of interest. This enables the study of enhancement patterns and provides important functional information for the diagnosis. The method has already been successfully used in the study of other organs, such as breast, myocardium, spleen and liver [22]–[25].

CEUS with TIC allows representation of the kidneys’ wash-in and wash-out phases after intravenous administration of USCA. In our research, we studied the kidney perfusion using CEUS with TIC to measure various perfusion indexes and correlate the results with the pathological findings, in an attempt to identify pathologic patterns in the early stage of CIRD.

In the early stage of CIRD, the number of microbubbles entering renal cortex noticeably decreased, leading to a decrease of back scattering signals comparable with the progressive blood flow decline of renal cortex. At the same time, the spasm of the renal cortex increased the resistance to its perfusion. As a result, CEUS enhancement and perfusion rate of renal cortex, decreased. Kidneys of these dogs showed weekly delayed enhancement and perfusion in the renal perfusion curve. It took more time to reach the peak intensity. As shown in our experiment, the gradually increased changes of AUC, TTP, DPI, α, and the decrease of A value, were statistically significant (*p*<.05). Earliest significant changes happened after operation on DPI and TTP.

Color and Power Doppler sonography are the most frequently used imaging procedures to evaluate the renal perfusion noninvasively by measuring resistance and perfusion index. However, such techniques as CDFI do not depict vessels with a diameter of less than about 30 µm, and rely on the determination of the so-called resistance index (RI) in diagnosing kidney dysfunction [26]. The value of color and power Doppler sonography is still debatable because of its inherent limitations, such as a lack of sensitivity to slow flow and angle dependency. Also, they were susceptible to error resulting from the examiner dependence of the method. The contrast-enhanced ultrasonography examination, on the other hand, is independent for the examiner and yields valid results even under difficult anatomic conditions as in obese patients [12], [13]. In our experiment, Significant changes of PSV and RI were observed at 12 ∼14 weeks after operation. Later than that of the CEUS quantitative indexes. In summary, the conventional CDFI examination, including the determination of RI values, provided no reliable basis for the diagnosis of early stage of CIRD perfusion changes. Contrast quantitative indexes provided more sensitive and early perfusion changes of renal cortex in dog’s model.

The status of renal blood perfusion reflected the changes of kidney function. In our research, we compared perfusion indexes of renal cortex with clinical laboratory data. In the early stage of CIRD, significant changes were observed in blood perfusion indexes from 4∼7 weeks after operation, but changes in SCr and BUN occurred in 11 weeks. As it was described in the literature, during the decompensation period of renal dysfunction, the occurrence of a characteristic increase of SCr and BUN becomes apparent only when GFR decreases to 1/3–1/2 of normal level [27]. Therefore they are not sensitive indexes for monitoring the early changes of renal function. Instead perfusion indexes are more sensitive and dependent parameters in the diagnosis of early stages of CIRD.

### Conclusions

The results of this preliminary animal investigation indicate that quantitative analysis of CEUS may provide useful information on the different stages of kidney perfusion in the progression of CIRD.

Even though this study was performed on animals, and the results should also be confirmed in humans before definite conclusions are stated, it provided some experimental evidences for future applications of microbubble-based agents in CIRD diagnosis. This could open up new perspectives for the sonographic study of the kidney, both transplanted and native.
